# Tell me why: A scoping review on the fundamental building blocks of fMRI-based network analysis

**DOI:** 10.1016/j.nicl.2025.103785

**Published:** 2025-04-13

**Authors:** Z. van der Pal, L. Douw, A. Genis, D. van den Bergh, M. Marsman, A. Schrantee, T.F. Blanken

**Affiliations:** a Amsterdam UMC location University of Amsterdam, Department of Radiology and Nuclear Medicine, Meibergdreef 9, Amsterdam, the Netherlands; b Amsterdam UMC location Vrije Universiteit Amsterdam, Department of Anatomy and Neurosciences, Boelelaan 1117, Amsterdam, the Netherlands; c University of Amsterdam, Department of Psychological Methods, Nieuwe Prinsengracht 129B, Amsterdam, the Netherlands; d University of Amsterdam, Department of Clinical Psychology, Nieuwe Achtergracht 129, Amsterdam, the Netherlands

**Keywords:** Static undirected networks, Functional MRI, Functional connectivity, Network neuroscience, Graph theory, Connectome

## Abstract

Understanding complex brain-behaviour relationships in psychiatric and neurological conditions is crucial for advancing clinical insights. This review explores the current landscape of network estimation methods in the context of functional MRI (fMRI) based network neuroscience, focusing on static undirected network analysis. We focused on papers published in a single year (2022) and characterised what we consider the fundamental building blocks of network analysis: sample size, network size, association type, edge inclusion strategy, edge weights, modelling level, and confounding factors. We found that the most common methods across all included studies (n = 191) were the use of pairwise correlations to estimate the associations between brain regions (79.6 %), estimation of weighted networks (95.3 %), and estimation of the network at the individual level (86.9 %). Importantly, a substantial number of studies lacked comprehensive reporting on their methodological choices, hindering the synthesis of research findings within the field. This review underscores the critical need for careful consideration and transparent reporting of fMRI network estimation methodologies to advance our understanding of complex brain-behaviour relationships. By facilitating the integration between network neuroscience and network psychometrics, we aim to significantly enhance our clinical understanding of these intricate connections.

## Highlights

• We characterise building blocks in functional MRI network neuroscience.
• Pairwise correlations were most often used to define connections.
• Estimation of weighted networks and individual level networks was most common.
• Reporting of network estimation choices was often incomplete.
• We identify opportunities to integrate brain and behaviour networks.

## 1 Introduction

Investigation of the brain-behaviour relationship is central to clinical research, as it is becoming clear that clinical phenotypes cannot be understood entirely by investigating the brain or behavioural factors alone (Blanken et al., 2021). Instead, complex interactions between (neuro)biological, behavioural and environmental factors may underlie e.g., development and sustenance of psychiatric disorders and degeneration in neurological disorders (de Boer et al., 2021, Kendler, 2008). Network analysis provides an analytical framework to investigate such complex interactions, and is increasingly used at both the brain level (network neuroscience) and behavioural level (network psychometrics). However, it is important to note that the application of network analysis, including the underlying principles and methods that are used, differs between these two fields. To bridge the gap and integrate these fields using network science, a better understanding of the adoption of methodologies in each of the fields is necessary. In this review, we aim to characterise the application and adoption of network methodologies in network neuroscience, particularly functional magnetic resonance imaging (fMRI) data, being the most widely adopted imaging modality in network neuroscience.

Since its inception over two decades ago, network neuroscience has rapidly advanced, finding increasing application in both cognitive and clinical neuroscience (Bassett & Sporns, 2017). Using network analysis, the field aims to advance our understanding of brain network organisation and functionality. In fMRI-based brain networks, nodes constitute brain regions, whereas edges represent the (statistical) associations between activity patterns of these brain regions (also referred to as functional connectivity). Network analysis has allowed us to amalgamate two important organisational principles in the brain: segregation and integration (Sporns, 2013). The adoption of network analysis has allowed the field to investigate the intricate functional interactions in the brain, and holds potential to provide valuable insights into understanding and tackling the complexities of clinical conditions.

In the realm of fMRI-based network neuroscience methods, a wide range of network estimation approaches is available. This review focuses on static undirected network analysis, given its wide application and the challenges of capturing common practices in the rapidly developing dynamic approaches. However, all approaches involve a multitude of choices regarding methodology, such as functional connectivity type and edge inclusion criteria. Importantly, these choices are non-trivial, as they yield substantial influence on the interpretation of network findings. For instance, different association types (e.g., pairwise versus partial correlations) can result in markedly different network topologies, leading to substantially different conclusions about functional connectivity patterns. Furthermore, it is not straightforward how to determine which edges to include in the network. Being mindful of these choices and their impact on interpretations becomes vital, perhaps especially when moving beyond studying just the brain and drawing conclusions about behaviour and clinical phenotypes. Precisely because network neuroscience and network psychometrics apply different methodologies, (complementary) knowledge and insights have been obtained in both fields. Combining these insights and integrating methodologies from both fields will boost developments to connect brain-based and behaviour-based networks. In order to connect these fields, a comprehensive understanding of prevalent network estimation methodologies in fMRI-based network neuroscience is crucial.

As a first step, this review aimed to characterise the prevalence of widely adopted network estimation methods that are currently being applied in the subfield of network neuroscience concentrating on fMRI-based static undirected network analysis. We characterised what we consider the fundamental building blocks of network analysis: sample size, network size, association type, edge inclusion strategy, edge weights, modelling level, and confounding factors (see methods for definitions). We focus on these fundamental building blocks, because they represent universal choices that need to be made when performing network analysis and that may impact network inferences. Deconstructing the network estimation process into such universal choices facilitates direct comparison between network neuroscience and network psychometrics, highlighting the challenges that need to be addressed to integrate these fields. Subsequently, we illustrate how such methodological choices can impact interpretation when applied to clinical neuroscience questions. Moreover, we highlight some studies that used a different approach compared with most studies, to demonstrate what alternative approaches are also being used.

## 2 Methods

### 2.1 Search strategy

We selected a representative sample of 11 journals that frequently publish papers applying fMRI functional connectivity and/or network analyses, ranging from specific network neuroscience to broader (neuro)scientific journals: Biological Psychiatry, Brain, Brain Connectivity, Cerebral Cortex, Human Brain Mapping, Journal of Neuroscience, Nature Communications, Network Neuroscience, NeuroImage, NeuroImage Clinical, and the Proceedings of the National Academy of Sciences (PNAS). To ensure the selected papers were representative of current broadly adopted practices, all papers published in 2022 in these journals were accessed through the “advanced search” function on the website Web of Science on January 9th, 2023.

For each of the selected journals, the following query was conducted on Web of Science (example for Biological Psychiatry below, indicated in **bold**): (TI=(FMRI) OR TI=(functional neuroimaging) OR TI=(functional MRI) OR AB=(FMRI) OR AB=(functional neuroimaging) OR AB=(functional MRI) OR KP=(FMRI) OR KP=(functional neuroimaging) OR KP=(functional MRI)) AND (TI=(connectivity) OR TI=(network) OR TI=(connect*) OR TI=(graph) OR AB=(connectivity) OR AB=(network) OR AB=(connect*) OR AB=(graph) OR KP=(connectivity) OR KP=(network) OR KP=(connect*) OR KP=(graph)) AND SO=(**biological psychiatry**) AND PY=(2022).

### 2.2 Exclusion criteria

To uniformly characterise the fundamental building blocks of prevalent fMRI network estimation methodologies, we here focus on studies using static undirected network analysis. We therefore excluded studies that did not perform static undirected network analysis on fMRI data. Static undirected networks are networks in which temporal variations in connectivity patterns are not considered and the direction of the relations between nodes is unknown. This approach is prevalent in network neuroscience and differs substantially from other network analytical methodologies, like time-varying and directed network analysis, in the way the networks are estimated (e.g., time-varying Granger causality versus Pearson correlation). Moreover, papers were excluded if the research was performed on non-human or foetal subjects, or if exclusively simulated data was used. Finally, reviews, editorials, and *meta*-analyses were excluded from this review.

### 2.3 Selection process

The titles and abstracts were evaluated by six reviewers (AS, DB, LD, MM, TB, ZP) using the Rayyan tool for systematic literature reviews (Ouzzani et al., 2016). All reviewers were blinded to each other’s ratings and each paper was rated as “include”, “exclude” or “maybe” by two reviewers. After unblinding, any discrepancies between the reviewers were resolved by discussion and consensus. Next, full-text evaluation of the included papers was performed by an additional reviewer AG and any discrepancies and/or unclarities were discussed and resolved with AS, LD and ZP. For detailed information on the informed consent procedures used in the included studies, please refer to the original papers (for a list of the included papers, see https://github.com/Schrantee-lab/GIN_networks_review.git).

### 2.4 Extraction of the fundamental building blocks of network analysis

**Fig. 1:**
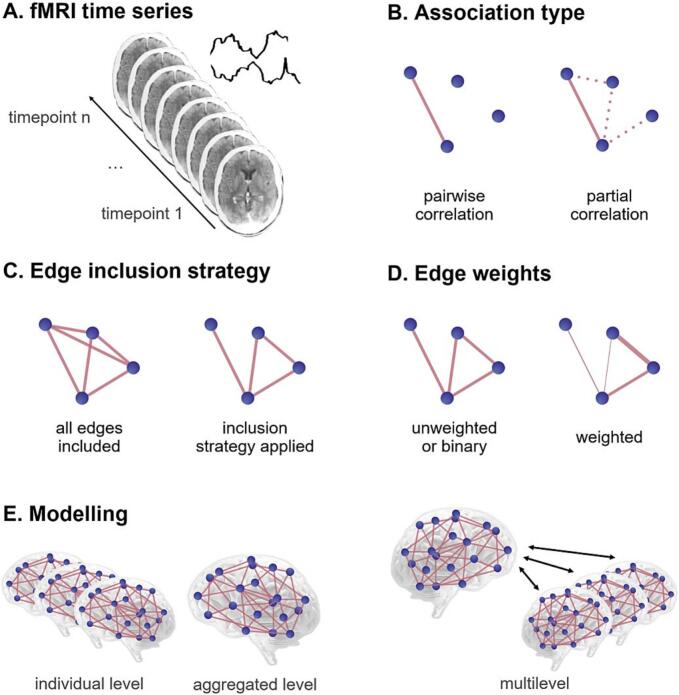
**Visual representation of functional magnetic resonance imaging (fMRI) time series and the fundamental building blocks of (brain) network analysis.** A) Functional connectivity is estimated based on the association of fMRI time series between predefined nodes. B) Association type: the method used to estimate the relation between two nodes in the network. C) Edge inclusion strategy: the strategy used to determine which edges to include in the network. D) Edge weights: whether the edges in the network were weighted or unweighted (binarised). E) Modelling: whether the network was modelled at the individual level or group level (aggregated modelling), or both (multilevel modelling).

To obtain an overview of the network estimation methodologies that are currently being applied in this subfield of network neuroscience, we extracted the following information from the selected papers (for visual representations and search terms, see Fig. 1 and Table 1):

● *Sample size*: the number of participants included for network analysis.
● *Network size*: the number of nodes (i.e., voxels, brain regions or networks of interest) that were included in the network.
● *Association type*: the method used to estimate the relation between two nodes in the network.
● *Edge inclusion strategy*: the strategy used to determine which edges to include in the network.
● *Edge weights*: whether the edges in the network were weighted or unweighted (binarised).
● *Modelling*: whether the network was modelled at the individual level or group level, or both. If group level networks were estimated, was the fMRI data concatenated prior to network estimation (i.e., aggregated modelling)? Or were both individual and group level networks estimated (i.e., multilevel modelling)?
● *Confounding factors*: whether confounding factors were taken into account. If any, were these confounding factors taken into account before, during, or after network estimation?

**Table 1:** The metrics extracted from the selected papers.

	**What is of interest?**	**Categories**	**Search terms**
**Sample size**	What is the sample size: number of participants (N)?		
**Network size**	What is the network size: number of variables (p)?		- Regions - Parcels - Variables - Nodes - Atlas - Region of interest (ROI)
**Association type**	How is the relationship between two nodes described?	- Pairwise correlation - Partial correlation - Regression - Other - Not specified	- Pairwise correlation - Full correlation - Partial correlation - Regression - Coefficient - Pearson - Spearman
**Edge inclusion strategy**	Were selection criteria applied to include an edge in the network?	- Yes - Thresholding - Regularisation - Etc. - No - Multiple - Not specified	- Thresholding - Binarize/binarise - Selection - Regularisation - (Model) comparison
**Edge weights**	Are the edges in the network weighted or unweighted (i.e., binarised)? If they are weighted, are negative edges included, or are negative edges absolutised or set to zero?	- Weighted - Unweighted - Multiple - Not specified	- Weight - Binarized/binarised
**Modelling**	How is the network estimated? At an individual or group level? If a group level network is estimated, is this done after concatenating the functional MRI data (i.e., aggregated modelling)? Or are both individual and group level networks estimated (i.e., multilevel modelling)?	- Individual - Aggregated - Multilevel - Not specified	- Individual - Aggregated over individuals - Hierarchical modelling - Multilevel modelling - Group level
**Confounding factors**	Were confounding factors taken into account? If any, were these confounding factors taken into account before, during, or after network estimation?	- Age - Sex/gender - Scan related - Biological/physiological - Clinical/psychological - Other - Multiple - Not specified	- Confounding/confounder - Covariate - Corrected/correcting - Adjusted/adjusting - Control/controlling - Nuisance

MRI = magnetic resonance imaging.

The extraction was performed by AG and ZP and any unclarities were discussed and resolved with AS and LD. Missing information was labelled as “not specified”. To visualise the results and provide an overview of common methodologies in fMRI-based network neuroscience, counts and proportions for each of the fundamental building blocks were summarised in pie charts and raincloud plots.

All analyses were performed using R (version 4.3.1, R Development Core Team) with the packages *cowplot* (version 1.1.1), *dplyr* (version 1.1.2), *ggplot2* (version 3.4.3), *ggpie* (version 0.2.5), *patchwork* (version 1.2.0), *readr* (version 2.1.5) and *scales* (version 1.2.1). The colour scheme was adapted from Frerebeau (2024) and the flat violin function was adapted from https://gist.github.com/dgrtwo/eb7750e74997891d7c20.

## 3 Results

### 3.1 Paper selection process

**Fig. 2:**
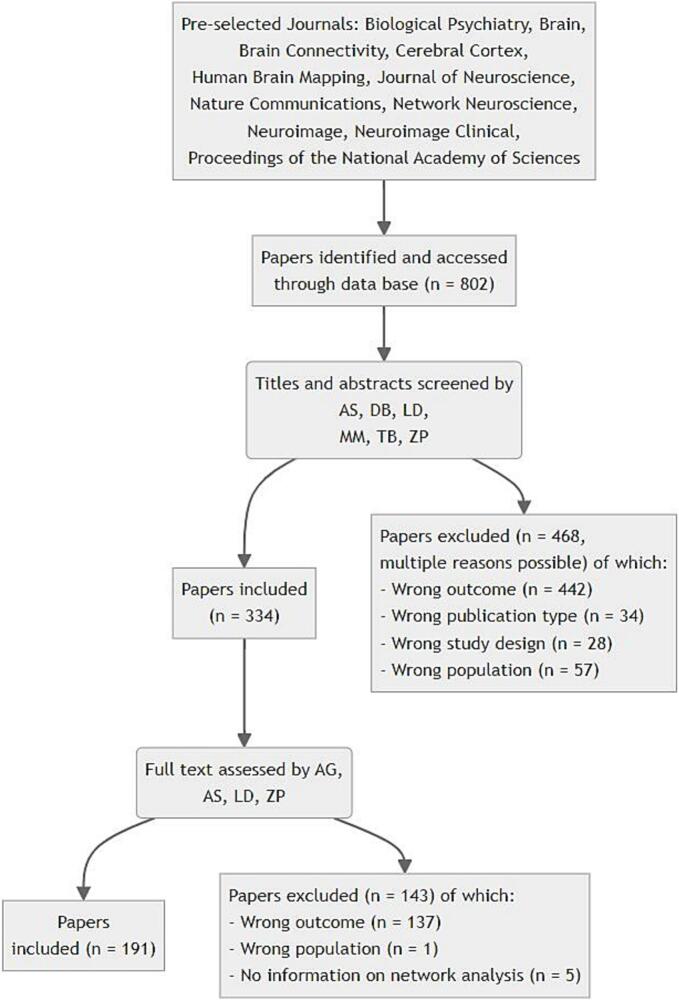
Overview of the systematic steps constituting the selection process.

A total of 802 papers were accessed from the selected journals and 191 papers were included in the analysis (for flow diagram and details, see Fig. 2 and Supplementary Table 1). A total of 611 papers were excluded (title and abstract screening: 468 papers; full text evaluation: 143 papers). The most common exclusion reasons were wrong outcome (e.g., task fMRI activity, seed-based analysis, effective or dynamic connectivity analysis, not fMRI; 579 papers), wrong population (e.g., non-human or foetal subjects; 58 papers), and wrong publication type (e.g., reviews; 34 papers). A total of 237 studies were excluded for not using static undirected network analysis (categorised as wrong outcome), demonstrating that while alternative approaches are not uncommon, static undirected network analysis constitutes a substantial focus of the reviewed literature. The total number of papers that was included from each journal is shown in Supplementary Table 2.

### 3.2 Fundamental building blocks of fMRI-based network analysis

**Fig. 3:**
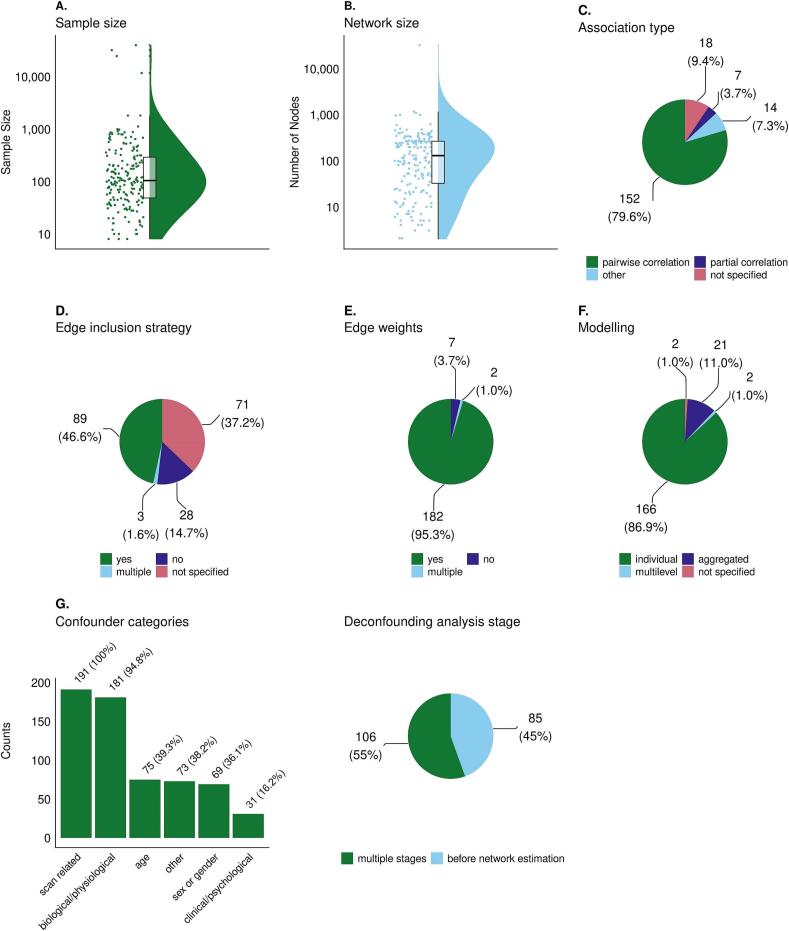
**Characterisation of the fundamental building blocks of static undirected network analysis using functional magnetic resonance imaging (fMRI) data.** A) Sample size. B) Network size. C) Association type. D) Edge inclusion strategy. E) Edge weights. F) Modelling. G) Confounding factors taken into account (left) at which stage of the analysis (right). Data are shown as raincloud plots with median (interquartile range), and pie and bar charts with counts (percentages). In panel B, one study was excluded from the visualisation, because the number of nodes was not specified (this was a voxelwise study). In panel G (left), note that nearly all studies (190, 99.5%) took into account multiple confounding factors.

Detailed results for each of the extracted metrics are shown in Fig. 3 and Supplementary Table 3. Across the 191 studies, the median sample size was 104 participants (interquartile range: 49–276; Fig. 3A). The median network size was 131 nodes (interquartile range: 33–268; Fig. 3B); one paper that performed voxelwise network analysis was excluded from this calculation because the number of nodes was not specified. Common combinations of the fundamental building blocks used by the reviewed studies include the use of pairwise correlations and thresholding to estimate weighted individual level networks (Supplementary Table 4). Notably, if a building block was not specified, particularly for edge inclusion strategies, this was often combined with very common choices for the other building blocks (e.g., pairwise correlations, weighted individual level networks), highlighting that there seem to be common methodological ‘pipelines’ that are likely widely adopted in the field.

#### 3.2.1 Association type

Most papers (152 papers, 79.6 %) estimated the associations between nodes using pairwise correlations, sometimes also referred to as full correlations (Fig. 3C). Partial correlations, which take into account all other nodes in the network, were used in seven papers (3.7 %). Furthermore, 14 papers (7.3 %) used alternative approaches to estimate the connections between nodes, such as regression (e.g., Freedberg et al., 2022, Thome et al., 2022, Yu and Fischer, 2022) or coherence measures (e.g.,Li et al., 2022, van Balkom et al., 2022, van Dijk et al., 2010). A subset of seven papers (3.7 %) used multiple approaches to estimate the associations between nodes. For instance, Strain et al. (2022) compared the use of pairwise correlations and covariance-based associations (i.e., unnormalised correlation, which retains sensitivity to systematic differences in the amplitude of fMRI signal fluctuations; Varoquaux et al., 2010a) and reported greater significance levels in correlation-based group level analysis compared with covariance-based associations. Moreover, in this study different results were obtained depending on the association type, age and (clinical) population (Strain et al., 2022). Finally, the remaining 18 papers (9.4 %) did not specify the association type used, or reported “correlation” as their association type without further specification.

#### 3.2.2 Edge inclusion strategy

Many papers (89 papers, 46.6 %) applied one or more strategies to select which edges to include in the networks (Fig. 3D). The most common approach was thresholding (68 papers, 35.6 %), with commonly used thresholds being a pairwise correlation p-value ≤ 0.05 (e.g., Falakshahi et al., 2022, Liu et al., 2022, Ren et al., 2022) or a density threshold where only a percentage of the strongest connections were retained (typically the top 10–30 % strongest connections; e.g., Kawabata et al., 2022, Pan et al., 2023, Wang et al., 2023). Six papers (3.1 %) used regularisation techniques, such as (graphical) least absolute shrinkage and selection operator (LASSO; e.g., Lund et al., 2022, Thome et al., 2022, Zhu and Qiu, 2022) or ridge-regression (Zhukovsky et al., 2023), to remove spurious edges from the network. Regularisation techniques penalise the fit of the model based on the model’s complexity in order to set spurious edges to zero. Moreover, two papers (1.0 %) retained only those edges that were present across multiple leave-one-out cross-validation rounds (Manglani et al., 2022, Wu et al., 2022). One study (0.5 %) used a minimum spanning tree approach to extract edges without applying a subjective threshold (Douw et al., 2022). Only 26 papers (13.6 %) constructed networks without any edge selection, creating fully connected networks. Notably, a large proportion of the studies (71 papers, 37.2 %) reported no information about which edges were included in the network.

#### 3.2.3 Edge weights

The use of weighted or unweighted networks was uniform across papers, as nearly all papers (182, 95.3 %) estimated weighted edges (Fig. 3E). Of these studies, five papers (2.6 %) replaced negative edges with their absolute values and four papers (2.1 %) set negative edges to zero. Furthermore, five studies (2.6 %) estimated unweighted networks, performing binarisation after thresholding. Finally, two studies (1.0 %) estimated both weighted and unweighted networks. Lee et al. (2022) estimated unweighted networks retaining only strong edges using a proportional threshold (5 %, 10 %, 30 %), as well as weighted networks with negative connections set to zero. Zhang et al. (2022) constructed unweighted networks after thresholding (p < 0.05/30135, Bonferroni-corrected), as well as absolutised weighted networks. Both studies used a different approach to evaluate network topology. Zhang et al. (2022) calculated different network measures from the unweighted and weighted networks, whereas Lee et al. (2022) calculated the same network measures from the weighted and unweighted networks and reported that these measures differed.

#### 3.2.4 Modelling: individual, aggregated, multilevel

Network modelling was mostly performed at the individual level (166 papers, 86.9 %; Fig. 3F). In 21 studies (11.0 %), the preprocessed fMRI data was concatenated and a group level network was estimated (i.e., aggregated network modelling). In addition, two studies (1.0 %) performed multilevel modelling. For instance, one study proposes a three-level multilevel model to predict individual functional connectomes from clinical characteristics, in which they use a machine learning approach to estimate the network at the voxel-level and region-level, and then impose sparsity on the network given the clinical characteristics across a range of clinical phenotypes (Morris et al., 2022). The second study combined functional and structural MRI data in a three-way parallel group-independent component analysis to generate group level as well as individual level networks (note that in this approach the data are first decomposed into functionally connected networks at the voxel level; Qi et al., 2022). Finally, two studies (1.0 %) did not specify the level at which networks were modelled.

#### 3.2.5 Confounding factors

All studies accounted for one or multiple confounding factors (Fig. 3G). A total of 85 studies (44.5 %) took confounding factors into account before network estimation only, for instance during data acquisition by matching participant groups or during fMRI data preprocessing. The remaining 106 studies (55.5 %) corrected for confounding factors at multiple stages of the analysis. Here, the most common approach was to correct for confounders before and after network estimation (102 studies, 53.4 %). Two studies (1.0 %) performed deconfounding before and during network estimation, and two studies (1.0 %) performed deconfounding before, during and after network estimation. The four studies that also corrected for confounders during network estimation used partial correlations (2 papers, 1.0 %), regression (1 paper, 0.5 %) or multiple approaches (pairwise correlation, partial correlation, tangent-space embedding; 1 study, 0.5 %) to estimate the associations between nodes. Deconfounding after network estimation was typically performed by including confounding factors as covariates or regressors of no interest when making statistical network inferences.

(Nearly) all studies corrected for scan related factors (e.g., motion; 191 studies, 100 %) and biological/physiological factors (e.g., cardiac and respiration related artefacts; 181 studies, 94.8 %) prior to network estimation. In addition, motion related measures, such as framewise displacement and the derivatives of the time series computed by obtaining the root mean square variance across voxels (DVARS), were commonly taken into account during the statistical analysis following network estimation (35 studies, 18.3 %). Moreover, age and sex or gender were commonly considered in the studies reviewed here. Age was accounted for before network estimation (10 studies, 5.2 %; e.g., by matching participant groups), during network estimation (1 study, 0.5 %), after network estimation (46 studies, 24.1 %), or both before and after network estimation (14 studies, 7.3 %). Sex or gender was taken into account before network estimation (9 studies, 4.7 %; e.g., by matching participant groups), after network estimation (47 studies, 24.6 %), or both before and after network estimation (8 studies, 4.2 %). Finally, other confounding factors that were commonly considered were clinical/psychological factors (e.g., primary or comorbid diagnosis, disease severity, task performance and medication use; 31 studies, 16.2 %), study site (in case of multicentre studies; 19 studies, 9.9 %), and education (24 studies, 12.6 %).

## 4 Discussion

This review aimed to determine how frequently different methodologies are currently being applied in static undirected network analysis using fMRI data, by characterising the fundamental building blocks of network estimation approaches. In this discussion, we will elaborate on how awareness of these choices and their implications is essential for meaningful synthesis of results within the fMRI-based network neuroscience field. This awareness becomes even more critical when connecting the field of network neuroscience to network psychometrics. In clinical studies of complex brain-behaviour relationships, this connection often centres on linking network topology to clinical research outcomes, such as diagnostic markers or treatment strategies.

Our findings revealed substantial commonalities in the selection of association type, edge inclusion strategies, edge weights and modelling approach that studies applied. However, it should be noted that a considerable number of studies reported incomplete or insufficient information on the methods used and choices made when estimating brain networks. This was particularly striking for edge inclusion strategies, which were not specified in over a third of the studies.

In the next sections, we will hone in on some important considerations and their implications. Although sample size, network size, and the method used to define the nodes in the network are important factors to consider when performing (brain) network analysis, these topics have been extensively discussed in literature (e.g. Bryce et al., 2021, Deschwanden et al., 2024, Domhof et al., 2021, Eickhoff et al., 2018, Fornito et al., 2010, Gong et al., 2018, Helwegen et al., 2023, Korhonen et al., 2021), and will therefore not be discussed in detail here.

### 4.1 Association type

Most studies reviewed here estimated the relations between nodes using pairwise correlations, which quantify the linear relationship between blood-oxygen-level-dependent (BOLD) activity of two nodes (i.e., brain regions; Aertsen et al., 1989). A high pairwise correlation value is thought to indicate in-phase coupling between regions (implying cooperation and integration of these regions), whereas a value of (nearly) zero is thought to indicate no linear relationship (e.g., implying decoupling of these regions). However, pairwise correlations cannot distinguish whether the association between two nodes is direct or whether it is influenced by other nodes in the network (Kim et al., 2015). The widespread use of pairwise correlations in fMRI-based brain network analyses may lead to over- or underestimation of the importance of particular interactions, because the possible influence of other nodes in the network is not considered. Partial correlations have been proposed as an alternative that alleviates this issue by quantifying the linear relationship between neural activity of two nodes *given all other nodes in the network* (Marrelec et al., 2006). In the case where two brain regions (A and B) are connected through a third brain region (C; A − C − B), the network would yield a connection between A and B in the pairwise correlation network but not in the partial correlation network. While this difference between the two networks is methodologically sound, it may lead to different clinical inferences: based on the pairwise correlation network we may conclude that a change in brain region A may be associated with a change in brain region B, but we would not draw this conclusion based on the partial correlation network. Thus, this methodological choice can have profound effects on our conclusions and, when not properly considered or reported, can hamper integration of study results from clinical studies, such as in *meta*-analyses.

Empirical and simulation studies on whether the use of pairwise or partial correlations in the fMRI literature is optimal are contradictory, with some studies favouring partial correlations (Cai et al., 2020, Cheng et al., 2012), pairwise correlations (Abraham et al., 2017) or showing no preference for either (Dadi et al., 2019). Moreover, certain network properties that are commonly calculated to describe network topology may be artificially inflated or reduced depending on the association type that is used (Zalesky et al., 2012). Taken together, there is no one optimal choice for association type, highlighting that the choice should be consciously made and elaborated upon based on the research question, also in combination with the characteristics of the studied population (Cassidy et al., 2018, Matkovič et al., 2023, Strain et al., 2022).

Finally, alternative association types provide additional insights compared to conventional correlation-based methods. One study compared the use of pairwise correlations with covariance-based associations (Strain et al., 2022). Covariance-based associations are sensitive to systematic differences in the amplitude of BOLD signal fluctuations, which is coupled with physiological measures like cerebral blood flow and glucose metabolism (Marchitelli et al., 2018, Wang et al., 2021b). Indeed, this alternative association type uncovered differential associations with age and clinical populations that were not captured using pairwise correlations (Strain et al., 2022). This empirical example illustrates that alternative association types require distinct interpretation, as they capture unique aspects of the data, recognising that each method provides unique information on brain network interactions that underlie clinical phenotypes.

### 4.2 Edge inclusion and weights

When estimating static undirected networks from fMRI data, at first all possible edges are included, constructing a fully connected network. However, it is important to realise that correlations between activity time series of different brain regions are prone to association, even in the absence of “real” functional connections, for instance due to measurement noise. Therefore, various approaches have been developed to reduce spurious connections in the network. The most commonly used method is thresholding, which retains only the connections above a certain threshold value and sets all other connections to zero. Thresholding is an effective method to exclude weak edges and obtain sparse network models, which can be helpful when the aim is to identify particular connections or (sub)networks that can be of interest for further research. Furthermore, the “backbone” of the network, i.e., the strongest connections and most well-connected nodes, have been shown to be most important for behavioural outcomes (Smith et al., 2023).

A downside of focusing on strong connections, is that the impact of weaker connections on global network structure may be overlooked, especially in research on heterogeneous phenotypes such as people with psychiatric disorders. Organisation of strong and weaker connections at the whole-brain level could differentially contribute to subtle interindividual variation in brain network topology (Gallos et al., 2012, Santarnecchi et al., 2014). Strong and weaker connections might reflect different properties of brain network topology, with strong connections generally representing prevalent short and long interhemispheric connections, and weaker connections usually reflecting long-distance connections between and within hemispheres (Santarnecchi et al., 2014). This means that focusing on strong connections potentially limits complete understanding of the complex interactions in the brain underlying clinical phenotypes. Consequently, selecting a threshold is a non-trivial task, with a variety of available approaches (e.g., selecting a uniform threshold value or retaining only a certain percentage of strongest edges in the network) that can yield a different number of edges in a network (i.e., network density). Since network structure and density influence the measures describing network topology, the choice for a particular thresholding approach may also alter the subsequent interpretations and conclusions drawn from the data. For example, when estimating individual networks, using a uniform threshold across all subjects may lead to different network densities and can therefore yield group differences in network properties that reflect differences in global network density rather than differences in network organisation (Jalili, 2016, van den Heuvel et al., 2017). Proportional thresholding, on the other hand, is more sensitive to inclusion of spurious edges, particularly in low-density networks, if the networks differ in global functional connectivity strength (Hallquist and Hillary, 2018, van den Heuvel et al., 2017, van Wijk et al., 2010). As a way to deal with this, it has been suggested to apply multiple thresholds or to conceptualise network properties as a function of a range of threshold values (Bassett et al., 2012, Bullmore and Bassett, 2011, Tu et al., 2024).

Alternative approaches include regularisation and only including edges that are present across multiple cross-validation rounds (e.g., Lund et al., 2022, Manglani et al., 2022, Thome et al., 2022, Wu et al., 2022, Zhu and Qiu, 2022). Regularisation techniques, such as LASSO, use data-driven rather than fixed thresholds. A less conventional, but arguably more principled approach is to perform regularisation after projecting the network onto the tangent-space of the Riemannian manifold (Barachant et al., 2013, Chen et al., 2010, Varoquaux et al., 2010b). This allows for efficient and robust analysis of fMRI-based networks, although some challenges should also be considered (e.g., the need for a reference covariance matrix; for an extensive discussion, see Pervaiz et al., 2020). Another edge inclusion strategy is the minimum spanning tree, which is a subgraph of the fully connected network that connects all nodes while minimising the sum of the edge weights (Douw et al., 2022, Stam et al., 2014). This approach yields sparse networks without using a subjective threshold, however important local connections may be neglected since cycles or clusters are not allowed (Jalili, 2016). Different strategies employ different assumptions about the “true” network underlying the data (e.g., minimum spanning tree assumes no isolated nodes in the network). Here too, the choice for a particular edge inclusion strategy affects the resulting network and should therefore be taken into account when interpreting the estimated network structure.

Even after determining which edges to include in the network, it can still be challenging to interpret connections in terms of network topology and importance. Most of the studies reviewed here constructed weighted networks. However, as illustrated above, the resulting network may differ substantially depending on the edge inclusion strategy that was applied. Moreover, edge weights may be subject to factors related to MR data acquisition, such as region-dependent variations in signal-to-noise ratio and motion (Fornito et al., 2016). As a result, subcortical regions are frequently excluded from brain networks due to their inherently low signal-to-noise ratio. An alternative is to estimate an unweighted (i.e., binarised) network, assigning edges the value zero (not present) or one (present; Hahn et al., 2019). Binarisation can help to gain insight into overall network architecture (which edges are present), irrespective of the strength of the individual connections. However, binarisation is applied after some form of edge selection and therefore is still sensitive to the influence of different edge inclusion strategies (i.e., binarisation strategies; Jalili, 2016).

The inclusion and meaning of negative edges is another topic of debate in the fMRI network literature (e.g., Zhan et al., 2017). Most studies that we reviewed here included positive as well as negative edges in the network, but there is no consensus on how negative connections should be interpreted (Murphy & Fox, 2017). Negative edges could play a distinct role in information processing (Parente et al., 2018), possibly reflecting NMDA-activation in cortical inhibition (Anticevic et al., 2012). Alternatively, negative edges in brain networks may stem from artefacts and correction for global signal changes (i.e., global signal regression; Anderson et al., 2011, Ibinson et al., 2015, Murphy and Fox, 2017, Saad et al., 2012). Notably, calculation of network properties may not be possible or requires adaptation if the network contains negative connections (Rubinov & Sporns, 2011). For these reasons, some studies choose to remove negative connections from the network by setting them to zero or using absolutisation. However, setting negative edges to zero disregards their influence on network topology, and replacing negative edges with their absolute values provides information only about strength, and no longer about directionality, of the connections between brain regions.

Clinical phenotypes may be associated with alterations in the number of connections that are present in the network, as well as their connection strength (Hallquist & Hillary, 2018). In order to assess both, one could consider estimating both weighted and unweighted networks, and compare results of different edge inclusion strategies. This was done by two studies reviewed here (Lee et al., 2022, Zhang et al., 2022). Alternatively, Bayesian network estimation approaches allow for concurrent evaluation of the strength of links, as well as whether links are present. For example, the Bayes factor can separate evidence for presence, evidence for absence, and lack of evidence for an effect (e.g., Dienes, 2014, Keysers et al., 2020). However, Bayesian analyses may be computationally heavy and time-consuming depending on the sample and network size (Sekulovski et al., 2023).

### 4.3 Modelling: individual, aggregated, multilevel

Most of the studies that we reviewed constructed separate networks per individual (i.e., individual network modelling), allowing for investigation of (changes in) network organisation at an individual level. Network properties calculated for each individual’s network can also be used in a group level analysis, providing insight into differential network organisation across populations. Alternatively, group level networks can be estimated by concatenating the fMRI data prior to network construction (i.e., aggregated network modelling). Similar to the other fundamental building blocks discussed above, the method used for data aggregation can impact the topology and stability of the resulting network (Cho et al., 2021). Considering that the human brain shows anatomical and functional similarities across individuals, group-average networks have been found to provide an adequate representation of an individual's brain network organisation (Seitzman et al., 2019). However, neither individual nor aggregated network modelling account for the simultaneous presence of shared information across individuals and interindividual differences in network organisation. Network topological measures calculated from individual or aggregated networks provide complementary information about brain organisation and functionality across individuals. Insights from both individual and aggregated network estimation approaches are essential for a comprehensive understanding of the complex interactions in the brain underlying clinical phenotypes and identifying potential subpopulations with distinct connectivity patterns, which are important goals of fMRI network studies.

Multilevel network modelling may provide a solution to disentangle individual and group network topology, by simultaneously estimating networks at the individual level and the group level. The individual network can be contrasted against the group model, providing insights into interindividual differences in network topology (Seitzman et al., 2019). Such interindividual variations in network organisation are very common (Gordon et al., 2017, Gratton et al., 2018, Mueller et al., 2013, Seitzman et al., 2019), especially in frontal and temporo-parietal cortical regions associated with higher-level functioning. Multilevel modelling can also uncover subgroups of stable network traits within a population (Seitzman et al. 2019), which is of interest for relating clinical phenotypes to variations in network organisation. Furthermore, multilevel network modelling allows for evaluation of network segregation and integration across multiple levels (e.g., individual and group networks, or brain and behaviour network; Fan et al., 2023, Wang et al., 2019), which has been proposed to be especially powerful for evaluating brain-behaviour relationships (Wang et al., 2021a). Such developments in multilevel network estimation and characterisation are still ongoing, and some challenges should be overcome. For instance, multilevel network modelling typically requires specification of the groups for which to model the group level network (e.g., patients versus controls), and it can be challenging to assign different individuals to a single group (e.g., in the situation where patients have multiple diagnoses). In addition, in case of large interindividual differences, the group level network may not be representative for the individuals and thus complicated to interpret. Furthermore, it should be noted that, besides multilevel network modelling, alternative approaches exist that take into account both individual and group level variation. For example, group-informed independent component analytical methods first estimate group level networks and subsequently use these as a reference to construct individual networks (Du and Fan, 2013, Iraji et al., 2023).

### 4.4 Confounding factors

The studies reviewed here showed substantial overlap in terms of deconfounding approaches. The most common confounds that studies corrected for were scan related factors (e.g., motion), physiological/biological factors (e.g., physiological noise), age, and sex or gender. Notably, most studies applied multiple approaches to correct for confounding factors. For example, motion effects were typically regressed out of the fMRI signal prior to network estimation, and motion related measures like framewise displacement and DVARS were also commonly included as covariates when making statistical network inferences following network estimation. The purpose of this approach is to correct for any residual motion effects that might remain after the initial motion correction performed before network estimation.

Only very few studies took confounding factors into account during network estimation. This aligns with our finding that most studies used pairwise correlations to estimate the edges in the network. In contrast to use of partial correlations and regression approaches, pairwise correlations are unable to take into account additional (confounding) factors when estimating the network (Kim et al., 2015, Marrelec et al., 2006). It is crucial to recognise that the resulting network and subsequent interpretation may differ depending on which confounding factors are taken into account at which stage(s) of the analysis.

### 4.5 Relations between building blocks and key underlying assumptions

Common combinations of the methodological building blocks used by the studies reviewed here include the use of pairwise correlations and thresholding to estimate weighted individual level networks. While some of these building blocks can be selected independently of one another (e.g., the choice to perform individual, aggregated or multilevel network modelling can be made independently from the selection of the other building blocks), there may also be dependencies between the different building blocks. For example, estimating a network using partial correlations typically yields smaller edge weights than when using pairwise correlations, since partial correlations take into account the influence of all other nodes/connections in the network. The same threshold value will likely lead to the inclusion of less connections in a network estimated with partial correlations versus pairwise correlations. If the edges are then binarised using the same threshold value to construct an unweighted network, the resulting networks will include different edges. Therefore, selecting appropriate network estimation methodologies for the research question at hand requires careful consideration, as well as an understanding of the assumptions underpinning different approaches.

While it is not feasible to provide guidance on optimal methodological choices for the wide range of research questions, we here try to clarify some key assumptions underlying widely adopted choices for the fundamental building blocks discussed in this review. For association type, use of pairwise correlations assumes that the relation between two regions is linear and is not influenced by other connections in the network. When selecting which connections to include in the network (edge inclusion strategy), thresholding approaches assume that weaker connections are not of interest and should be excluded to reduce spurious connections. In contrast, including all edges in the network assumes that all connections have a relevant contribution to network topology and that the influence of spurious connections (e.g., due to measurement noise) is limited/negligible. In a weighted network, stronger connections are considered to contribute more to network topology, whereas in an unweighted network (i.e., after binarisation) all connections are considered to have equal importance. Finally, estimating separate networks per individual (i.e., individual network modelling) does not consider the presence of a group level network that may be present across participants, whereas a group level network (i.e., aggregated network modelling) has the underlying assumption that the network is constructed across a representative and/or homogenous population.

### 4.6 Clinical interpretation and network psychometrics

Ultimately, an important goal of fMRI network studies is to link brain network connectivity to relevant behaviours in health or disease (Bassett & Bullmore, 2009), aiming to identify biomarkers and informing personalised treatment strategies. Using association type as an example again, we can illustrate how the choice between partial and pairwise correlations can influence clinical interpretation. When selecting brain regions for non-invasive neuromodulation techniques like transcranial magnetic stimulation (TMS), partial correlations may identify direct network connections between brain regions that arguably have a more targeted effect on symptomatology. Pairwise correlations also reflect indirect associations and thereby may reveal regions that are linked to multiple areas, albeit directly or indirectly − potentially important for predicting side effects of neuromodulation.

At the behavioural level, the complexity and importance of interactions between key entities, such as attitudes, behaviours, and symptoms, are increasingly recognised (Borsboom et al., 2021). This recognition has been accompanied by a surge in methodological developments in the field now known as network psychometrics (Isvoranu et al., 2022, Marsman and Rhemtulla, 2022). A critical example of the methodological advances that have been made within network psychometrics is seen in one of our fundamental building blocks: edge inclusion strategy. Within network psychometrics, the early datasets used for network estimation consisted of relatively few observations compared to the number of variables. For example, one of the earliest studies included 240 nodes and only 129 participants (Cramer et al., 2012). Such data characteristics required innovative ways to determine which edges were stable enough to include in the network. In this context, LASSO regularisation provides a viable approach (Tibshirani, 1996), as it can shrink certain edge weights to exactly zero, which reduces the number of estimated edges, leading to more interpretable and robust network results (Epskamp and Fried, 2018, van Borkulo et al., 2014). This is very similar to the thresholding approach commonly used in fMRI research, but unlike current thresholding approaches, LASSO learns the thresholding point from the data under certain constraints, such as maximising an information criterion (e.g., Chen & Chen, 2012). As such, it requires less predefined, subjective decisions regarding thresholding, which could be interesting to apply more often in fMRI network studies as well.

Another relevant innovation within network psychometrics is the use of a Bayesian approach (Marsman et al., 2022, Williams, 2021, Williams and Mulder, 2020). Using advanced Bayesian model-averaging techniques (Huth et al., 2023, Sekulovski et al., 2023), it is possible to quantify evidence for the inclusion of an edge and, critically, also evidence for the exclusion of an edge or the absence of evidence altogether (e.g., that there is too little information to detect the edge). This new approach in network psychometrics could fruitfully connect to dominant research questions on the sparsity of fMRI networks (Mumford & Ramsey, 2014).

Another example of why it is relevant to focus on these building blocks is illustrated by considering the modelling approach. In network neuroscience, networks are usually estimated for each individual (i.e., individual network modelling), whereas in network psychometrics group-average networks are more common (i.e., aggregated network modelling). To bridge these fields, a critical step is to develop methodologies that explicitly link individual and group-average networks. For instance, techniques such as hierarchical modelling, subject-specific parameter estimation within group models, or hybrid approaches that combine both individual and group level information could facilitate meaningful comparisons between network neuroscience and network psychometrics.

We have previously suggested and discussed methods to construct brain-behaviour networks (Blanken et al., 2021), which we briefly reiterate here. In the multilayer network approach (Brooks et al., 2020, Simpson-Kent et al., 2023), brain and behavioural networks are estimated separately and connected through inter-layer associations, resulting in a network with both within- and between-layer links. The integrated network method (Hilland et al., 2020) combines brain and behavioural data into a single, unified network. Lastly, the network-based regression approach (Bathelt et al., 2022) regresses out the effects of all other nodes in the behavioural network to obtain residuals, which are then used to identify brain correlates for each behavioural node. It is important to note that these all require specific network estimation methods that may not be fully developed. Alternatively, multivariate approaches reducing the parameter space can be employed to provide additional insight, like canonical correlation analysis (CCA) and partial least squares (PLS), which aim to capture shared information between brain, behavioural and environmental factors in the form of latent variables (see e.g., Vieira et al., 2024).

### 4.7 Reporting practices

Different network estimation approaches each have their own advantages and pitfalls, therefore careful consideration at each step in the network estimation process is imperative. Nonetheless, these decisions and their rationale are often not (completely) reported, at least not in the papers we reviewed. While it is challenging to determine researchers' motivations without direct insight into their decision-making processes, factors like established practices, availability of computational and training resources, interpretability, and publication bias could potentially play a role. However, the observed patterns of homogeneous methodological choices hint at systemic influences, potentially driven by factors like ease of use, computational efficiency and publication trends. Future research exploring why researchers make certain methodological choices is needed, including thorough qualitative approaches (e.g., using surveys or interviews with researchers).

The prevalence of missing methodological information in fMRI network analysis studies raises significant concerns for clinical interpretation, as well as research reproducibility and integration. Furthermore, incomplete reporting can hinder accurate replication of studies and impede *meta*-analyses, ultimately slowing scientific progress in the field. Implicit reporting (e.g., reporting “correlation” as association type, without further specification) can still lead to ambiguity, since individuals with different (scientific) backgrounds can have different underlying assumptions and therefore will come to different interpretation and conclusions. Insufficient reporting of methods is a known issue, and already in 2016 the widespread variability in reporting of resting-state fMRI processing methods was described (Waheed et al., 2016). Since then, the Committee on Best Practice in Data Analysis and Sharing (COBIDAS) of the Organization for Human Brain Mapping (OHBM) developed a guideline for reporting of methodologies regarding data acquisition, processing and network estimation (Nichols et al., 2017). Moreover, preregistration can help to address issues related to the incomplete reporting of methods by requiring researchers to specify their analysis plans in advance (Flannery, 2020, Nichols et al., 2017, Poldrack et al., 2008). The adoption of such standardised reporting guidelines and preregistration practices would enhance reproducibility and transparency of fMRI network analyses and facilitate more robust comparisons across studies.

Beyond methodological development, concrete steps toward integration of network neuroscience and network psychometrics include establishing a joint research agenda that prioritises cross-disciplinary methodological advancements, designing coordinated data collection efforts that allow for direct comparisons across levels of analysis, and developing unified reporting standards to ensure consistency in the interpretation of network topology changes in clinical studies. These initiatives would provide a structured framework for translating network insights into clinically meaningful applications, ultimately advancing the utility of network-based approaches in clinical studies.

### 4.8 Limitations

Some limitations of the current work should be considered. Firstly, we only included studies that performed static undirected network analysis on fMRI data and were published in a specific set of journals in one particular year. We acknowledge that methodological choices in alternative functional connectivity approaches (i.e., not static undirected network analysis) may be different from the fundamental building blocks discussed here. Secondly, other factors prior to the network estimation can also strongly influence results and their subsequent interpretation. For instance, differences in data quality (resulting from MR hardware and software parameters) and choices regarding preprocessing and analysis of the MRI data can be influential. Concerning the latter, the decision to include or exclude certain processing steps (e.g., global signal regression), or the rigour of nuisance regression (e.g., motion) can influence the resulting time series from which the brain networks are estimated (for an extensive discussion on this, see Andellini et al., 2015, Botvinik-Nezer et al., 2020, Gavrilescu et al., 2008, Luppi et al., 2024, Wanger et al., 2023).

## 5 Conclusion

We reviewed the fundamental building blocks of static undirected network analysis using fMRI data. We discussed the implications of the selection of different network estimation methodologies in order to create awareness about the choices that need to be made when estimating brain networks from fMRI data, as well as their implications for interpretation of the results. We highlight that a single “correct” approach to uncover the “true” network topology of the brain does not exist: network construction methods should be chosen in relation to the question at hand. We emphasise that complete and transparent reporting is imperative for open science and reproducibility, as well as interpretation of network results. With this review, we hope to facilitate integration between network neuroscience and network psychometrics when studying complex brain-behaviour relations, providing a foundation for development of targeted, more personalised treatment strategies.

## CRediT authorship contribution statement

**Z. van der Pal:** Conceptualization, Formal analysis, Investigation, Writing – original draft, Writing – review & editing. **L. Douw:** Writing – review & editing, Investigation, Funding acquisition, Formal analysis, Conceptualization. **A. Genis:** Writing – review & editing, Writing – original draft, Investigation, Formal analysis. **D. van den Bergh:** Writing – review & editing, Investigation, Formal analysis, Conceptualization. **M. Marsman:** Writing – review & editing, Investigation, Funding acquisition, Formal analysis, Conceptualization. **A. Schrantee:** Writing – review & editing, Writing – original draft, Supervision, Investigation, Funding acquisition, Formal analysis, Conceptualization. **T.F. Blanken:** Writing – review & editing, Writing – original draft, Supervision, Investigation, Funding acquisition, Formal analysis, Conceptualization.

## Funding

This project was supported by an Amsterdam Public Health − Personalised Medicine grant and an 10.13039/501100019541Amsterdam Brain and Cognition grant. MM was supported by the 10.13039/501100000780European Union (ERC, BAYESIAN P-NETS, 101040876). Views and opinions expressed are, however, those of the authors only and do not necessarily reflect those of the 10.13039/501100000780European Union or the European Research Council. Neither the European Union nor the granting authority can be held responsible for them. AS was supported by a 10.13039/501100003246Dutch Research Council Veni grant (016.196.153).

## Declaration of competing interest

The authors declare that they have no known competing financial interests or personal relationships that could have appeared to influence the work reported in this paper.

## Data Availability

The data extracted from the reviewed papers and the code used to synthesise the results of this review are available at https://github.com/Schrantee-lab/GIN_networks_review.git.
