# Supplementary material for: Tell me why: A scoping review on the fundamental building blocks of fMRI-based network analysis
Source: Neuroimage Clin. 2025 Apr 13;46:103785. doi: 10.1016/j.nicl.2025.103785 (PMC12264222; doi:10.1016/j.nicl.2025.103785)
Supplement: Supplementary Data 1 [file mmc1.pdf]

Tell me why: A scoping review on the fundamental building blocks of fMRI-based network analysis

Supplementary Materials

Supplementary Table 1. Papers excluded during title and abstract screening and full text evaluation. A paper can be assigned multiple reasons for exclusion.

**Reason**	**Title and abstract screening** ***N***	**Full text evaluation** ***N***
Wrong outcome	442	137
Analysis method (e.g., dynamic connectivity, effective connectivity, ReHo, fALFF)	119	118
Acquisition (not functional MRI)	50	7
Task activity	187	12
Wrong publication type	34	-
(Systematic) review	10	-
Editorial	1	-
Background article	3	-
Wrong study design (no empirical data)	28	-
Wrong population (non-human, foetuses)	57	1
No information on network analysis	-	5
Total excluded	468	143

fALFF = fractional amplitude of low-frequency fluctuations; MRI = magnetic resonance imaging; ReHO = regional homogeneity.

Supplementary Table 2. Papers extracted and included per journal for characterisation of the fundamental building blocks of network analysis.

Journal	Extracted<br>*N*	Included<br>*N* (%)
Biological Psychiatry	14	1 (7%)
Brain	27	8 (30%)
Brain Connectivity	24	8 (33%)
Cerebral Cortex	156	44 (28%)
Human Brain Mapping	153	43 (28%)
Journal of Neuroscience	36	5 (14%)
Nature Communications	36	3 (8%)
Network Neuroscience	22	4 (18%)
NeuroImage	225	43 (19%)
NeuroImage Clinical	83	24 (29%)
Proceedings of the National Academy of Sciences	14	5 (36%)

Supplementary Table 3. Complete results of the extracted values for each of the fundamental building blocks of network analysis.

Fundamental building block	Categorisation	*N* (%)
Association type	Pairwise correlation	152 (79.6)
	Partial correlation	7 (3.7)
	Other	11 (5.8)
	Various approaches	7 (3.7)
	Regression	3 (1.6)
	Coherence	2 (1.0)
	Permutation entropy	1 (0.5)
	Accordance of activation peaks	1 (0.5)
	Not specified	18 (9.4)
	Not further specified correlation	14 (7.3)
	Not specified at all	4 (2.1)
Edge inclusion strategy	Yes	89 (46.6)
	Thresholding	68 (35.6)
	Regularisation	6 (3.1)
	Negative edges set to 0	4 (2.1)
	Cross validation	2 (1.0)
	Minimum spanning tree	1 (0.5)
	Normalised entropy and high amplitude co-fluctuations	1 (0.5)
	Averages of multiple runs for a single participant	1 (0.5)
	Correlation with behavioural outcomes	2 (1.0)
	Edges involving nodes of interest	1 (0.5)
	Various approaches	3 (1.6)
	No	28 (14.7)
	Not applied	26 (13.6)
	Not applicable	2 (1.0)
	Multiple (with and without)	3 (1.6)
	Not Specified	71 (37.2)

Edge weights	Yes	182 (95.3)
	Absolutised	5 (2.6)
	Not absolutised	177 (92.7)
	No	7 (3.7)
	Not applied	5 (2.6)
	Not applicable	2 (1.0)
	Multiple (weighted and unweighted)	2 (1.0)
Modelling	Individual	166 (86.9)
	Aggregated	21 (11.0)
	Multilevel	2 (1.0)
	Not Specified	2 (1.0)
Confounding factors*	Age	75 (39.3)
	Sex or gender	69 (36.1)
	Scan related	191 (100)
	Biological/physiological	181 (94.8)
	Clinical/psychological	31 (16.2)
	Other	73 (38.2)

* Note that nearly all studies (190, 99.5%) took into account multiple confounding factors.

Supplementary Table 4. Combinations of the fundamental building blocks used by the reviewed studies. Data are presented as the number of studies (%).

	Association type				Edge inclusion strategy						Edge weights		
	Pairwise correlation	Partial correlation	Other	Not specified	No	Yes (thresholding)	Yes (regularisation)	Yes (other)	Multiple	Not specified	Weighted	Unweighted	Multiple
**Edge inclusion strategy**													
No	20 (10.5%)	2 (1.0%)	4 (2.1%)	2 (1.0%)									
Yes (thresholding)	54 (28.3%)	1 (0.5%)	5 (2.6%)	8 (4.2%)									
Yes (regularisation)	1 (0.5%)	2 (1.0%)	1 (0.5%)	2 (1.0%)									
Yes (other)	14 (7.3%)	1 (0.5%)	0 (0.0%)	0 (0.0%)									
Multiple	3 (1.6%)	0 (0.0%)	0 (0.0%)	0 (0.0%)									
Not specified	60 (31.4%)	1 (0.5%)	4 (2.1%)	6 (3.1%)									
**Edge weights**													
Weighted	145 (75.9%)	7 (3.7%)	12 (6.3%)	18 (9.4%)	26 (13.6%)	62 (32.5%)	6 (3.1%)	14 (7.3%)	3 (1.6%)	71 (37.2%)			
Unweighted	5 (2.6%)	0 (0.0%)	2 (1.0%)	0 (0.0%)	2 (1.0%)	5 (2.6%)	0 (0.0%)	0 (0.0%)	0 (0.0%)	0 (0.0%)			
Multiple	2 (1.0%)	0 (0.0%)	0 (0.0%)	0 (0.0%)	0 (0.0%)	1 (0.5%)	0 (0.0%)	1 (0.5%)	0 (0.0%)	0 (0.0%)			
**Modelling**													
Individual	135 (70.7%)	5 (2.6%)	12 (6.3%)	14 (7.3%)	23 (12.0%)	56 (29.3%)	3 (1.6%)	15 (7.9%)	3 (1.6%)	66 (34.6%)	158 (82.7%)	6 (3.1%)	2 (1.0%)
Aggregated	13 (6.8%)	2 (1.0%)	2 (1.0%)	4 (2.1%)	5 (2.6%)	11 (5.8%)	2 (1.0%)	0 (0.0%)	0 (0.0%)	3 (1.6%)	21 (11.0%)	0 (0.0%)	0 (0.0%)
Multilevel	2 (1.0%)	0 (0.0%)	0 (0.0%)	0 (0.0%)	0 (0.0%)	1 (0.5%)	1 (0.5%)	0 (0.0%)	0 (0.0%)	0 (0.0%)	1 (0.5%)	1 (0.5%)	0 (0.0%)
Not specified	2 (1.0%)	0 (0.0%)	0 (0.0%)	0 (0.0%)	0(0.0%)	0 (0.0%)	0 (0.0%)	0 (0.0%)	0 (0.0%)	2 (1.0%)	2 (1.0%)	0 (0.0%)	0 (0.0%)
